# A Low-Cost, Easily Accessible Simulation Model for Microsurgery Training

**DOI:** 10.29252/wjps.8.2.265

**Published:** 2019-05

**Authors:** Bilsev Ince, Mehmet Emin Cem Yildirim, Mehmet Dadaci

**Affiliations:** Necmettin Erbakan University, Meram Faculty of Medicine, Department of Plastic, Reconstructive and Aesthetic Surgery, Konya, Turkey

**Keywords:** Microsurgery training, Simulation model, Anastomosis

**DEAR EDITOR**

Although the number of microsurgical operations increase day by day, it is still difficult to learn due to the requirement of specific subset of skills. Unlike traditional surgery, experience with microsurgical instruments and performing with microscope is very important for success.^1^ Generally, microsurgical training programs involve silastic tubes and practice on experimental animals or cadavers.^2^^,^^3^ The use of experimental animals and cadavers in microsurgical training is believed to be a gold standard. However, the use of a simulation model to gain experience with microscope and microsurgical instruments may be more ethically acceptable. 

We would like to present a simulation model that we use in basic microsurgical training program of our clinic, which is very cheap and can be reached easily in every hospital. The model can be created using fingers of a surgical glove, a glue, an injector needle and two injector needle caps. The needle cap is cut in half and the distal parts of the caps are brought together. Then injector needle is passed from one to another (Figure 1a). Fingers of the glove are wrapped and glued on both sides of the caps. (Figure 1b). This model provides both vessel-like softness to microsurgery trainees, and the needle passing through the center stabilizes this model. 

**Fig. 1:**
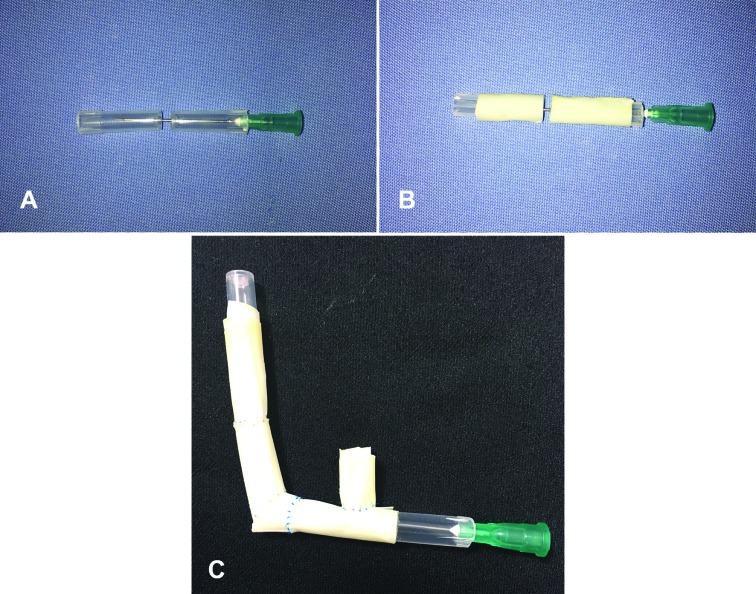
**A)** The needle cap is cut in half and the distal parts of the caps are brought together. Then injector needle is passed from one to another. **B)** Fingers of the glove are wrapped and glued on both sides of the caps. **C)** The view of the end to side anastomosis

Also, it provides help to develop hand-eye coordination and to gain experience with the use microsurgical instruments under the microscope. This model can be used with 7.0, 8.0, 9.0 and 10.0 suture materials. It can also be used as a simulation model in end-to-end, end-to-side anastomosis and cases with vascular diameter mismatch (Figure 1c). In conclusion, after training on this model that costs only 40 cents, microsurgery trainees can learn easily on the animals.

## CONFLICT OF INTEREST

The authors declare no conflict of interest.
